# Attitudes and behaviors concerning self-medication practices among adult urban population in Croatia: a cross-sectional study

**DOI:** 10.3389/fphar.2026.1864658

**Published:** 2026-07-21

**Authors:** Vedrana Aljinović-Vučić, Marin Vučić, Renata Jurišić Grubešić

**Affiliations:** 1 Faculty of Medicine, University of Rijeka, Rijeka, Croatia; 2 School of Medicine, University of Zagreb, Zagreb, Croatia

**Keywords:** community pharmacists, drug use, family physicians, over-the-counter drugs, prescription drugs, self-medication

## Abstract

**Background and Aim:**

Drug utilization worldwide is often associated with practicing self-medication of prescription or non-prescription drugs. Irresponsible behaviors in this field can lead to a variety of health risks. Assessing the extent of self-medication in population contributes to guiding decisions on future interventions to improve it. This study aimed to investigate self-medication patterns and attitudes of adult urban inhabitants in Croatian cities Zagreb and Rijeka.

**Methods:**

A cross-sectional study was conducted from April to May 2025 in urban community pharmacies involving 402 adult customers who filled out the questionnaire on their self-medication behaviors and attitudes, adverse events related to self-medication, and practices of storing medications at home. The data was analyzed using descriptive statistics. The Chi-square test was used to estimate association between sociodemographic features and self-medication practices.

**Results:**

Most commonly self-medicated drugs were analgesics-antipyretics by 94.2% participants. Antimicrobials were self-medicated in 29.4% of cases, and anxiolytics in 24.0%. Higher educational levels were associated with stronger inclination to self-medicate in general (p = 0.034). Most commonly, respondents acquired over-the counter drugs for self-medication in pharmacies (75.3%). Women bought over-the-counter drugs and reported adverse drug reactions more frequently (p = 0.033 and p = 0.037, respectively). 77.5% of participants kept drugs in a designated area (home pharmacy), and 24% of participants could not estimate if drugs they kept at home were potentially toxic.

**Conclusion:**

The prevalence of reported self-medication was rather high, even with prescription drugs such as antibiotics and anxiolytics. Educational activities and practical interventions should be implemented to increase the awareness of potential risks of inappropriate self-medication and to strengthen responsible behavior in this aspect with family physicians and community pharmacists having the key role in the process.

## 1 Introduction

The consumption of drugs globally is often connected with self-medication of both, prescription and over-the counter drugs. According to the World Health Organization (WHO), self-medication refers to the use of medicinal products by individuals to treat conditions they diagnosed by themselves, as well as the sporadic or regular use of drugs prescribed earlier by a physician for chronic or recurring diseases or symptoms (World Health Organization, 2000).

Responsible self-medication is considered a component of self-care, which the WHO broadly defines as actions taken by individuals or communities to promote and maintain health without the direct involvement of healthcare professionals (HCPs) (World Health Organization, 2013). When practiced responsibly, meaning the use of over-the-counter (OTC) medicines as directed in the patient information leaflet—self-medication can offer benefits such as taking care of minor ailments by patients themselves and reducing the pressure on healthcare systems (World Medical Association, 2022; Bertsche et al., 2023).

However, irresponsible self-medication involves behaviors such as using prescription drugs without medical advice, self-diagnosing and independently selecting treatment, or reusing previously prescribed therapies without consulting a physician. It also includes taking medicines based on the advice of non-healthcare individuals (e.g., family or friends) or recommending or giving medications to others without medical guidance (Ruiz, 2010).

Irresponsible use of medicines without professional supervision can lead to overdosing, harmful drug interactions, and adverse effects that may require urgent medical intervention (Ylä-Rautio et al., 2020; de Oliveira et al., 2014; Frei et al., 2010; Munir et al., 2022; Schmiedl et al., 2014). Even over-the-counter drugs can be dangerous when misused (de Oliveira et al., 2014; Frei et al., 2010; Schmiedl et al., 2014; Serper et al., 2016; Rajanayagam et al., 2015). Moreover, self-medication can delay accurate diagnosis and proper treatment, leading to disease progression which can be particularly dangerous in the case of serious illnesses (Ylä-Rautio et al., 2020). The misuse of certain medications, especially antimicrobials, contributes to the global problem of antimicrobial resistance (AMR), a major public health threat (World Health Organization, 2023). These issues extend beyond personal health, posing a considerable socioeconomic burden due to the increased healthcare costs required to address complications arising from inappropriate medicine use (Antimicrobial Resistance Collaborators, 2022).

Despite the potential dangers, self-medication is widely practiced across all age groups (Nazari et al., 2024; Zheng et al., 2024; Amenta et al., 2022; Kłoda et al., 2024; Tarciuc et al., 2020; Jensen et al., 2014). Children are often self-medicated by parents or caregivers (Kłoda et al., 2024; Tarciuc et al., 2020; Jensen et al., 2014), and it is common practice even among healthcare professionals and healthcare students (Aşut et al., 2025; Kim et al., 2025; Cotobal-Calvo et al., 2025; Mohammed et al., 2021; Galán Andrés et al., 2021; Alduraibi et al., 2022; Kokabisaghi et al., 2024; Benameur et al., 2019; Mustafa et al., 2023). Although HCPs generally possess the necessary knowledge regarding medication use and associated risks and often display more responsible attitudes, they still engage in self-medication at high rates, including with antibiotics (Benameur et al., 2019; Mustafa et al., 2023).

The COVID-19 pandemic further amplified self-medication practices. In some cases, this was a practical response, as overwhelmed healthcare systems benefited when patients managed mild symptoms independently. However, in many instances, self-medication during the pandemic was inappropriate and driven by fear and misinformation (Mejia et al., 2023; Zhang et al., 2021; Makowska et al., 2020). Some individuals used prescription drugs such as chloroquine, hydroxychloroquine, and azithromycin without medical oversight, hoping to treat or prevent COVID-19 (Mejia et al., 2023). The pandemic also encouraged drugs stockpiling at home, a behavior linked to higher rates of self-medication, not only during the pandemic but beyond (Amenta et al., 2022).

Although numerous studies have addressed different aspects of self-medication, more comprehensive data is needed to fully understand current practices and public attitudes. These insights are essential for guiding future policies and strategies aimed at improving self-medication behaviors.

The primary aim of our study was to assess the prevalence of self-medication among urban setting adult individuals frequenting community pharmacies and to investigate associated factors.

## 2 Materials and methods

### 2.1 Study design and setting

A voluntary anonymous questionnaire-based cross-sectional study was conducted to assess self-medication practices and attitudes of adults (aged 18 and above) in two bigger Croatian cities, Zagreb and Rijeka. Zagreb is Croatian capital and Rijeka is the capital of County Primorje-Gorski Kotar. According to the 2021 census, population of Zagreb was 767.131 while the population of Rijeka was 107.964 (total of 875.095). Calculation to assess the number of participants to be included in the study was performed utilizing standard single proportion formula for sample estimation:

$$n=\left(Z^{2}\;\cdot \;p\left(1‐p\right)\right)\;/\;e^{2}$$

With 95% confidence interval (Z = 1.96), 5% margin of error (e) and expected prevalence 50% (p), the required sample size was estimated to be 384. The survey was performed between April 3rd and 7 May 2025 at community pharmacy chain.

### 2.2 Ethical considerations

This study was performed in accordance with relevant institutional and ethical standards, as well as with the 1964 Helsinki declaration and its later amendments or its comparable standards. It was approved by Ethics Committee of the community pharmacy chain (“Pablo” pharmacies, Ethical approval number: 0001-2025/25-04). Informed consent was obtained from all individual participants of the study. Participants received written information outlining the study’s anonymous and voluntary nature, along with its objectives, the type of data being collected, and instructions for completing the structured questionnaire. Participants were informed that they could withdraw at any time without any consequences. Those who consented proceeded to complete the questionnaire.

### 2.3 Data collection

Adults (age 18 and above) attending participating pharmacies were randomly invited to participate in this voluntary and anonymous survey. The survey was self-administered for participants to complete it autonomously. Nevertheless, pharmacists at the participating pharmacies were available to assist participants in case any part of the survey required clarification.

### 2.4 Study questionnaire

The questionnaire used as the main survey instrument was developed *ad hoc* for this study based on previously published studies and specifically adapted to align with the objectives of this study (Aljinović-Vučić et al., 2005; Aljinović-Vučić et al., 2025). To verify the clarity and validity of the questionnaire extensive literature research was performed and experts from the field of pharmacy practice and research, pharmacology and public health were consulted. According to their inputs questionnaire was corrected and edited to improve the clarity to ensure comprehensible questions and robust methodology. To ensure questionnaire was appropriate and applicable to this study, we asked eight experts selected based on their experience and/or academic record in pharmacology, clinical pharmacy, pharmacy practice, pharmaceutical research and public health to validate it. Content Validity Ratio (CVR) was assessed to be 0.91 (minimum CVR value of 0.75 was considered acceptable). Additionally, Content Validity Indexes (CVI) for relevance and clarity were assessed. Item validity scores (I-CVI) above 0.71 were considered acceptable and scores ≥0.78 were considered excellent for items on both scales. For Scale Content Validity Index (S-CVI/UA) the universal agreement method was used for both scales (relevance and clarity) and was 0.89 for each of them. No additional psychometric validation (including internal consistency or reliability testing) was performed since the questionnaire comprised of categorical descriptive items rather than latent-scale constructs. Consequently, conventional reliability measures (such as Cronbach’s α) were not applicable, as the instrument did not contain multi-item scales intended to assess underlying psychometric constructs. Instead, it consisted of standalone categorical variables. To further assess the clarity, validity and understandability of the questionnaire a pilot study was performed on 40 individuals. Inclusion criteria were the same as in the main study. Since the feedback did not result in any changes, the data from the pilot study was included in the main study.

The questionnaire collected information on the following topics: 1) Main sources of information on drug use; 2) Practice of self-medication (i.e., use of drugs without consultation with a healthcare professional); 3) The most common illnesses or symptoms prompting self-medication; 4) Main reasons for practicing self-medication; 5) Frequency of self-medication by drug category; 6) Main sources from which participants obtained self-medicated drugs; 7) Attitudes toward self-medication; 8) Experiences of adverse events related to self-medication; 9) Type of adverse events encountered; 10) Whether medical assistance was needed for resolving adverse events that were consequence of self-medication; 11) Whether adverse events were reported and to whom; 12) Whether participants had a designated place at home for storing medicines (e.g., home pharmacy); 13) Location of the home pharmacy within the household; 14) Awareness of potentially toxic drugs stored at home; 15) Availability of medications at home to children under the age of 14. The questionnaire consisted of multiple-choice questions requiring participants to select a single response from the options provided. If participants selected the option “other” they could have presented their own answer instead of the offered options. The question about attitudes to self-medication offered the opportunity to write an explanation for every selected option. The frequency of practicing self-medication by drug category was assessed by filling out the table where participants could select the frequency of use for particular group of drugs with options being “never”, “occasionally” and “regularly”. Self-medication was defined as the use of medications without prior consultation with a healthcare professional regarding the indication, dosage, or duration of treatment for symptoms recognized by the individual, and without professional recommendation.

In addition to self-medication related data, general demographic information was collected, including age, gender, the highest level of education, and whether the participant was a healthcare professional or student (e.g., physician, dentist, pharmacist, veterinarian, or a healthcare student).

### 2.5 Statistical analysis

The data were analyzed using IBM SPSS Statistics for Windows, Version 25.0. (Armonk, NY: IBM Corp). Sociodemographic variables were treated as independent predictors, while behaviors and attitudes related to self-medication were treated as dependent variables. Since the study was observational and survey-based, analysis was designed to identify associations rather than establish causality. Descriptive, inferential and multivariate statistical methods were used for data analysis. Data were summarized as counts and percentages. Descriptive statistics including frequency distributions and percentages were used to analyze sociodemographic data. The Shapiro-Wilk test was used to assess normality of distribution. Differences between subgroups were tested using the independent samples t-test. The Chi-square tests were used to analyze differences in frequencies between groups and were applied to examine identification of sociodemographic characteristics and potential contributing factors for self-medication behaviors or attitudes. Multivariate binary logistic regression was conducted to identify which demographic factors or attitudes significantly increased the likelihood of engaging in self-medication. The Nagelkerke R square test was used to assess the explanatory power of the model, while the Hosmer–Lemeshow test was applied to evaluate model fit. Statistical significance was set at p < 0.05 for all analyses.

## 3 Results

### 3.1 Sociodemographic data

A total of 402 valid questionnaires were collected. Demographic information was provided by 399 respondents, of whom two-thirds were female (66.2%) and one-third male (33.8%). All participants were adults aged over 18. Almost half of the respondents reported they had a college degree or higher while 47.1% had high school degree and the percentage of participants with elementary school or below was 3.8%. Out of 397 respondents who answered the question on being healthcare professional (HCP), 13.4% identified themselves as HCPs, including physicians, pharmacists, doctors of dental medicine, nurses, doctors of veterinary medicine, and students of healthcare studies. Sociodemographic data are presented in Table 1.

**TABLE 1:** Sociodemographic characteristics of survey participants.

Sociodemoghraphic characteristics	N	%
Gender		
Male	135	33.8
Female	264	66.2
Total	399	100.0
Age		
18–29	69	17.3
30–39	56	14.0
40–49	92	23.1
50–59	81	20.3
60–69	64	16.0
≥70	37	9.3
Total	399	100.0
The highest level of education		
Elementary school or below	15	3.8
High school	188	47.1
College, university and graduate studies	196	49.1
Total	399	100
Healthcare professional		
No	344	86.6
Yes	53	13.4
Total	397	100.0

### 3.2 Self-medication practices

When asked directly about self-medication practices, more than half of participants (60.8%) reported engaging in self-medication. However, when asked about specific symptoms or conditions for which they self-medicated, 82.3% listed at least one despite some initially did not disclose their engagement in self-medication. Commonly self-treated conditions included general pain, fever, headache, cold/flu, allergies, gastrointestinal issues, musculoskeletal discomfort, diarrhea, constipation, bloating, and cough. A statistically significant association was observed between a greater tendency to self-medicate and higher educational attainment (χ^2^ (2) = 6.77, p = 0.034, Cramér’s V = 0.13), the observed effect size was small. No significant difference was found between HCPs and non-HCPs regarding general engagement in self-medication (p = 0.442).

### 3.3 Types of drugs used for self-medication

The most commonly self-medicated drugs for which respondents disclosed at least occasional self-medication were analgesics-antipyretics (94.2%), followed by vitamins and minerals (81.5%) and cough medicines (77.4%). Details by pharmacotherapeutic drug group are shown in Table 2.

**TABLE 2:** Use of different pharmacotherapeutic drug groups for self-medication.

Pharmacotherapeutic group of drugs	N (participants who self-medicate)*	%
Analgesics-antipyretics	357	94.2
Vitamins and minerals	296	81.5
Cough medicines	267	77.4
Antacids	190	55.6
Topical dermal drugs	175	52.7
Allergy drugs	143	42.8
Topical ocular drugs	117	35.7
Antimicrobials/antibiotics	96	29.4
Anxiolytics	79	24.0
Antihypertensives/antilipemic drugs	48	14.5
Antidiabetic drugs	20	6.1

* Percentages were calculated based on the number of respondents who answered the question for each pharmacotherapeutic group.

**FIGURE 1:**
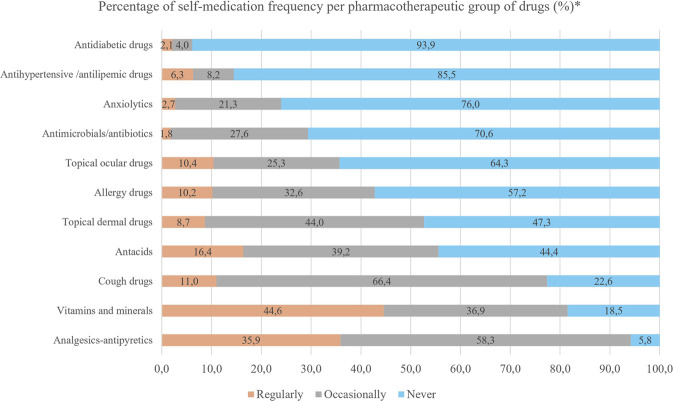
Frequency of self-medication by drug group. *Percentages were calculated based on the number of respondents who answered the question for each pharmacotherapeutic group.

Concerning the frequency of self-medication with particular groups of drugs, most respondents disclosed they practice self-medication with analgesics-antipyretics at least occasionally (94.2%). More than three-quarters of participants reported self-medication with cough drugs (77.4%). Antacids were used for self-medication in more than half of participants (55.6%) similarly to topical dermal medications (52.7%). Medications for allergy and topical ocular medications were used for self-medication in more than 1/3 of respondents (42.8% and 35.7% respectively). Among prescription-only medicines, antimicrobials were used for self-medication at least occasionally by 29.4% participants, anxiolytics by 24.0%, antihypertensives by 14.5%, and antidiabetics by 6.1%. The frequency of self-medication by specific therapeutic category is presented in Figure 1.

Anxiolytic and antihypertensive/antilipemic self-medication was found to be more common among participants with lower education levels (χ^2^ (4) = 13.46, p = 0.009, Cramér’s V = 0.14) and χ^2^ (4) = 17.66, p = 0.001, Cramér’s V = 0.16 respectively). The observed effect sizes ranged from small (V = 0.10) to small-to-moderate (Cramer’s V = 0.16).

Men practiced self-medication with drugs for treating allergy (χ^2^ (2) = 6.41, p = 0.041, Cramér’s V = 0,14) and vitamins/minerals (χ^2^ (2) = 16.06, p < 0.001, Cramér’s V = 0,21) less frequently than women with small-to-moderate effect sizes. HCPs were more likely to use antimicrobials/antibiotics for self-medication, the strength of the association was small to moderate (χ^2^ (2) = 12.81, p = 0.002, Cramér’s V = 0.20).

Age-related differences showed greater use of analgesics-antipyretics among respondents aged 30–39 (χ^2^ (10) = 21.84, p = 0.016, Cramér’s V = 0.17). Antacid medications were regularly used for self-medication among participants aged 30–39, and the least used in group aged 18–29 (χ^2^ (10) = 20.83, p = 0.022, Cramér’s V = 0.18). Drugs for allergy were significantly more frequently used for self-medication among participants aged 18–29 and 30–39 while the oldest group (aged 70 and over) practiced self-medication of allergy medications the least frequently (χ^2^ (10) = 27.02, p = 0.003, Cramér’s V = 0.20). The effect sizes were small to moderate.

The observed effect sizes ranged from small (Cramer’s V = 0.10) to small-to-moderate (Cramer’s V = 0.20), indicating their practical magnitude was modest. However, these associations reached statistical significance, implying that detected differences were unlikely to have occurred by chance and the association was likely real.

Since reported levels of self-medication with antimicrobials and anxiolytics were rather high concerning both pharmacotherapeutic groups are prescription-only drugs, binary logistic regression was performed on outcomes: self-medication: “Yes” (“regularly” or “occasionally”) or “No” (“never”). Regression analysis identified one significant predictor for antibiotic self-medication: being an HCP (OR = 2.750, 95% CI: 1.346–5.620, p = 0.006). HCPs had 2.7 times higher probability for self-medication with antimicrobials. Concerning anxiolytics, it identified age over 50 as predictor of self-medication: participants aged 50–59, 60–69, and 70 and over had higher probability for self-medication of anxiolytics for 3.5 times (OR = 3.549, 95% CI: 1.251–10.067, p = 0.017); 5.0 times (OR = 5.007, 95% CI: 1.685–14.872, p = 0.004); and 3.7 times (OR = 3.711, 95% CI; 1.009–13.640, p = 0.048), respectively.

### 3.4 Sources of advice about drugs use

**FIGURE 2:**
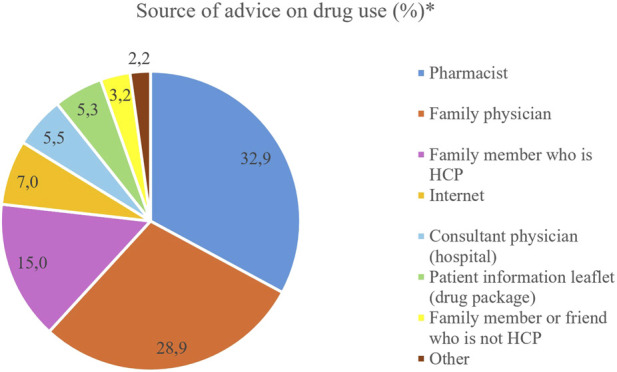
Source of advice on drug use. *Calculated on total number of respondents who answered this question (N = 401).

Most participants cited healthcare professionals as their main source of advice concerning drug use followed by family physicians and family member who was HCP. Participants with higher educational levels more often cited HCP family members as information sources, while those with medium educational levels primarily relied on physicians Men more frequently used the internet, whereas women relied on pharmacists (χ^2^ (7) = 33.17, p < 0.001, with a moderate effect size Cramer’s V = 0.29). Sources of advice on drug use are shown in Figure 2.

### 3.5 Reasons for self-medication

The most frequently cited main reason for self-medication was the confidence in personal ability to recognize symptoms (51.5%). Other reasons included: long waiting times at family physicians’ offices (17.4%); internet research (13.1%); an advice from non-HCP family/friends (8.4%). Some respondents who answered “other” offered their own reasons for self-medication including advice of a family member (or close person) who is an HCP; being an HCP themselves; being acquainted enough with symptoms/medicines and can estimate themselves whether self-medication is enough, previous advice received from HCP.

### 3.6 Sources of drugs

Most participants reported buying OTC drugs at pharmacies (75.3%), followed by using leftover prescribed medicines (12.6%) and obtaining medicines from family/friend (8.6%). No significant differences were found in drug sourcing across education or age groups, although more women reported buying OTC drugs (χ^2^ (3) = 8.93, p = 0.033, with a small effect size Cramér’s V = 0.16).

### 3.7 Attitudes towards self-medication

In aspect of attitudes towards self-medication, the answers were almost equally distributed among; positive attitude; practicing self-medication only in a situation which they recognize as necessary; negative attitude; and no defined attitude. HCPs were more likely to view self-medication positively, while non-HCPs more often had no clear opinion (χ^2^ (3) = 16.97, p = 0.001, with a small-to-moderate effect size Cramér’s V = 0.21). Negative or undefined attitudes were associated with reduced likelihood of engaging in self-medication (OR = 0.211, p = 0.01 and OR = 0.151, p = 0.002, respectively). Reasons for engaging in self-medication, sources of drugs used for self-medication, and attitudes on self-medication are listed in Table 3.

**TABLE 3:** Information on behaviors and attitudes regarding self-medication.

Behaviors and attitudes regarding self-medication	N	%
The main reason for engaging in self-medication		
I Believe that I can recognize the symptoms and disease by myself well enough and that I can choose the appropriate therapy	177	51.5
Waiting times in family practice offices are too long for me to wait to get medicines	60	17.4
I Find the relevant information about symptoms and treatments on internet	45	13.1
I Get the information from friends and family who are not HCPs	29	8.4
Other	28	8.1
I Find it inconvenient to ask for an advice in the pharmacy because of lack of privacy	5	1.5
Total	344	100.0
The main source of drugs which respondents use for self-medication		
Buying OTC drugs in pharmacy	281	75.3
Taking leftover drugs that were previously prescribed by physician	47	12.6
Using medicines from other family members or friends	32	8.6
Other	13	3.5
Total	373	100.0
The attitude on self-medication		
I Do not have any attitude about self-medication	104	26.7
Self-medication should be practiced only when necessary	103	26.4
Self-medication is positive and beneficial	96	24.6
Self-medication should be avoided because it can be dangerous	78	20.0
Other	9	2.3
Total	390	100.0

### 3.8 Self-medication and adverse drug reactions (ADRs)

Most participants (92.3%) reported never experiencing ADRs after self-medication. Only 1.6% of them required physician intervention because of such ADRs. Reporting rates were low (9.0%). Those who reported ADRs did so to family physicians (48.5%), pharmacists (39.4%), specialist physicians (9.1%), or national regulatory agency (9.1%). Information on experiences with ADRs associated with self-medication is shown in Table 4.

**TABLE 4:** Information on experiences with adverse drug reactions associated with self-medication.

Adverse drug reactions as consequence of self-medication	N	%
Experiencing ADRs after self-medication		
Yes	30	7.7
No	362	92.3
Total	392	100.0
The need for physicians’ intervention after experiencing ADRs		
Yes	6	1.6
No	362	98.4
Total	368	100.0
ADRs reporting		
Never reported	342	91.0
Reported	34	9.0
Total	376	100.0

Women reported ADRs more frequently (OR = 2.63, 95% CI 1.05–6.58). The association was statistically significant, χ^2^(1) = 4.35, p = 0.037, although the effect size was small (Cramér’s V = 0.11). No differences were found by educational level, age, or HCP status. Participants who experienced an ADR after self-medication listed mostly gastrointestinal disturbances (diarrhea vomiting, nausea, gastrointestinal pain and symptoms, stomachache, reflux, heartburn), followed by dermal symptoms (skin irritation or redness) and facial or tongue swelling.

### 3.9 Characteristics of storing medicines at home

Most respondents (77.5%) kept medicines in a designated area, 14.2% stored them in multiple locations, and 8.3% did not store medicines at home. Participants aged 50–59 were most likely to use a designated place, whereas those aged 18–29 showed tendency to store medicines across various locations (χ^2^ (10) = 28.17, p = 0.002, Cramér’s V = 0.19).

Among respondents, 14.5% (95% CI 11.0–18.0) reported having toxic medicines at home, while 61.5% (95% CI 56.7–66.3) denied presence of such medicines at home, and 24% were unsure (95%CI 19.8–28.2). HCPs more often correctly identified toxic medicines. Uncertainty was more prevalent among non-HCPs (χ^2^ (2) = 21.8, p < 0.001, Cramér’s V = 0.24). No differences were found by gender, age, or education.

In 57.9% of households, no children were present. In households with children under 14, medicines were accessible to children in 7.8% of cases. There was no significant difference in the presence of toxic medicines between households with or without children (p = 0.158). An association was observed between higher educational level and storing medicines out of reach of children under 14 years of age in the household, χ^2^ (4) = 12.9, p = 0.012, (Cramér’s V = 0.13) with small strength of the association. Characteristics of medicines storage at homes are presented in Table 5.

**TABLE 5:** Characteristics of drugs storage at home.

Characteristics of drugs storage	N	%
Drugs are kept		
At specific location (home pharmacy, medical cabinet)	307	77.5
Around the apartment (no specific location for storing drugs)	56	14.2
There are no drugs at home	33	8.3
Total	396	100.0
The location of the home-pharmacy/medical cabinet		
Living room	91	28.5
Bedroom	75	23.5
Kitchen	66	20.8
Bathroom	47	14.7
Hall	31	9.7
Other places (basement, ironing room, study room, pantry)	9	2.8
Total	319	100.0
Presence of potentially toxic drugs		
Yes	57	14.5
No	241	61.5
Unable to estimate	94	24.0
Total	392	100.0
Accessibility to children < 14 years		
There are no children <14 years in the household	228	57.9
Drugs are not accessible to children <14 years	153	38.8
Drugs are accessible to children <14 years	13	3.3
Total	394	100.0

## 4 Discussion

Self-medication is a globally present practice. Various studies were conducted with the aim of estimating different aspects of self-medication, but they applied different methodologies and were set in varying and specific contexts such as COVID-19 pandemic which impedes direct comparison among them. However, it has been shown previously that level of self-medication is rather high and still can be considered irresponsible in many aspects (Zheng et al., 2024; Tarciuc et al., 2020; Aşut et al., 2025; Galán Andrés et al., 2021; Alduraibi et al., 2022; Benameur et al., 2019; Mustafa et al., 2023; Makowska et al., 2020).

Participants of this study were adult city inhabitants who visited pharmacies during the period of the survey. Although when asked directly they disclosed self-medication practices in 60.8%, when asked about engagement in self-medication for various symptoms/diseases, 82.3% of respondents listed that they practice self-medication in cases of certain conditions. Symptoms or diseases listed as the reason for self-medication were usually mild which was in accordance with the finding that respondents disclosed confidence in recognizing the symptoms of the disease/condition as the reason for engaging in self-medication in more than a half (51.5%) of cases. This survey showed no significant difference in engagements in self-medication between respondents who were HCPs or those who were not. On the other hand, the association was observed between engaging in self-medication practices and higher educational level of participants (p = 0.034 Cramér’s V = 0.13), although with modest effect size. Previously published literature showed that people with higher educational level engage more in self-medication, possibly because they feel more confident in their knowledge and the ability to recognize if the condition requires HCPs attention or not (Nazari et al., 2024; Zheng et al., 2024; Du and Knopf, 2009). In our study a high percentage of the participants had high school degrees or higher (>95%). Since survey was performed in bigger Croatian cities it was expected that inhabitants would have higher educational level since those cities are administrative centers either of the country (Zagreb) or of the one of bigger regions/counties (Rijeka) with universities, hospital centers and industry which employ persons with higher degrees. Living in bigger cities where the network of healthcare centers and community pharmacies is more developed than in rural communities, could influence the extent of self-medication and patients’ engagement in it. Although city inhabitants might have better access to HCPs, in our survey as the second main most common reason for engaging in self-medication, participants listed “long waiting times in physicians’ offices” (17.4%) which corresponds to reasons listed as cause of engagement in self-medication in previously published literature (Lei et al., 2018; Aslam et al., 2024). In future research it would be interesting to compare if there is a difference between urban and rural settings in this aspect.

The drugs used for self-medication by the most respondents were analgesics-antipyretics in 94.2% of cases. These are the groups of drugs which previously had also been shown to be most commonly used for self-medication (Jensen et al., 2014; Ge et al., 2022; Martín-Pérez et al., 2016; Kıroğlu et al., 2022). This could be perceived as expected considering the finding that majority of the respondents disclosed practicing self-medication for treating conditions such as headache, pain in general, high temperature and musculoskeletal discomfort. It is important to notice that although many of analgesic-antipyretic drugs can have OTC status, some of them can also be prescribed by physicians and can pose a serious risk to person’s health when not used in an appropriate manner. This also applies to the other groups of drugs that were reported to be amongst most frequently used for self-medication such as antacids, topical dermatologic drugs, and drugs for allergy which can be both prescribed and obtained as OTC medications. Altogether, these findings confirm the need for increased awareness and understanding of possible risks of self-medication and the need for strengthening the education of lay persons.

Furthermore, finding information about symptoms and treatments on internet was listed as the third most common reason for practicing self-medication (13.1%). Considering the growth of reach and influence of digital media and information on internet, this number could still increase in the future. However, it is important that this finding is observed from both sides: 1) as possible threat, because there is a vast amount of fake information and incorrect content on social media and various platforms. Healthcare community as well as authorities should be aware of that to properly contain the proportions of its impact; 2) as possible opportunity for HCPs, regulators, pharmaceutical industry, and authorities to develop and implement regulations and interventions to ensure that content which digital media provide on diseases, symptoms, diagnostics and treatments is accurate, appropriate and controlled to avoid possible consequences of misinforming. In this context, there is also a need to consider development of telepharmacy and online consultations with pharmacists or chatbots to obtain advice on appropriate drug use. This feature is expected to be increasingly available and used in the future and represents an opportunity to guide patients in the direction of appropriate drug use and responsible self-medication in general. Therefore, it is important that pharmacies’ web pages as well as online pharmacies ensure this service is provided. On the other hand, it is crucial to warn patients about possible appearance of non-registered online pharmacies which could offer drugs that should not be purchased nor taken without HCPs supervision or drugs of lower quality. Patients should be advised to obtain drugs exclusively from registered online pharmacies which have a regulatory authorization to dispense medical products online, and to be cautious when obtaining medical products online in general.

Especially worrisome was reported rate of participants who disclosed engagement in the practice of self-medication with prescription medicines such as antimicrobials/antibiotics in almost 1/3 of cases (29.4%) which is in accordance with some other previously published studies (Nazari et al., 2024; Wang et al., 2018; Jifar et al., 2024). These behaviors need attention in at least two aspects: this shows increased availability of mentioned medications to patients (from whatever source, e.g., leftover medications, home pharmacies, sharing with friends or family) which they can use without HCPs’ supervision, and the existence of risks connected with the unsupervised use of prescription medications which can have even serious consequences. Additionally, when lay persons practice it, it is probable that they would engage in self-medication of antibiotics even when there is not real need for it such as in cases of viral infections, flu-like symptoms or even common cold which they could misdiagnose for a bacterial infection (Jifar et al., 2024; Arboleda Forero et al., 2023; Väänänen et al., 2006). All of that can lead to the growth of AMR and these behaviors show that people engage in self-medication practice without considering this issue. Although over previous years AMR development and the need to contain it through responsible use of antibiotics have been widespread topics, these findings show a strong necessity to further expand the awareness and education about it.

Furthermore, the finding that 24.0% of participants engaged in self-medication with anxiolytics at least sometimes, poses the reason for concern especially when the risk of dependence development as well as sedative potential of drugs in this pharmacotherapeutic group are considered. Since the study investigated urban population, this observation cannot be simply extrapolated to entire Croatian population. It warrants further research in this area to estimate the prevalence of antibiotic self-medication as well as self-medication in general, more accurately.

Although the level of self-medication was reportedly high, most participants claimed they predominantly obtained information about the proper use of drugs from HCPs (82.3%) with pharmacists (32.9%) and family physicians (28.9%) being listed as the primary source of information by most respondents. This could be used in future efforts to advise patients/lay persons and guide them in direction of appropriate self-medication in variety of ways such as medication revision, medication management, and giving more direct counselling on when and how to practice self-medication. These efforts should also include strengthening the awareness of primary HCPs in detecting if there is significant self-medication on-going and reacting in such cases.

Concerning attitudes on self-medication, participants who stated that self-medication was positive and beneficial as one of explanations for this particular attitude listed: “they could recognize symptoms and the disease themselves based on previous experience”; “they don’t waste time on waiting” in the doctors’ offices; “they could react faster and symptoms would go away faster”. The availability of OTC drugs also was among listed reasons. However, some of them disclosed reasons as “unavailability of physicians” and “physicians did not pay enough attention on analyzing their symptoms/diseases”. HCPs were found to be more positively oriented toward practicing self-medication than lay persons who were found not to have clear opinions in more cases. Although, among the reasons for having a negative attitude toward self-medication was the possibility of developing adverse drug reactions (ADRs), most study participants (92.3%) disclosed they had never experienced an ADR with only 1.6% of respondents who disclosed that ADR they experienced required physician’s intervention.

Storing medicines at home was previously shown to be one of the factors associated with the increased level of self-medication even with antibiotics (Wang et al., 2018). In our survey 91.8% of participants disclosed storing medications at home. More than three-quarters of respondents (77.5%) stored drugs at a designated area (home pharmacy). Among respondents 21.2% disclosed the source of drugs used for self-medication were leftover drugs previously prescribed to them or drugs taken from friends or family. It is possible that a part of antibiotics an anxiolytics used for self-medication were sourced among these drugs kept in households. This emphasizes the importance of management of home pharmacies and implies that more responsible and safe storage of medicines could add to overall decreasing of their inappropriate use. It is also connected with several risks such as possibility of accidental poisoning, using medications in a wrong manner or using expired drugs which consequently results in ineffective treatment. This is one of the activities in which primary HCPs could be more actively involved by encouraging patients to inspect their home drug supplies. Especially community pharmacists can intervene in this part by offering patients who are uncertain about drugs toxicity to bring drugs to the pharmacy for review and help them discard drugs which are not needed any more as well as the ones with past expiry dates.

Limitations of this study were that it was performed only in urban settings, and it would be useful to additionally investigate the practices and behaviors of rural population to get a more comprehensive view. Also, the questionnaire was not subjected to formal psychometric reliability testing, such as internal consistency assessment, because its items were categorical and independent rather than scale-based. However, content and face validity were supported through expert review and pilot testing. Nevertheles, these limitations do not diminsh relevance of this research and findings which can be compared to similar studies.

Results of this study show self-medication is spread across all groups regardless of some differences observed among them. There is a necessity for constant education of population in this aspect. It is especially needed to increase the awareness of possible risks when self-medication is inappropriate but also to implement educational activities to raise the level of knowledge of population on when to use self-medication and when to visit a physician. The role of pharmacist is particularly important when it comes to counselling patients on proper medication use as well as on when to visit a physician. Pharmacists and family physicians are primary HCPs who can discover if there is self-medication on-going and if the possibility of significant drug interactions exists. Pharmaceutical intervention could be particularly helpful in underserved communities where access to physicians is reduced due to underdeveloped healthcare networks such as rural communities or due to long waiting times in doctor’s offices as in cities. To increase awareness on appropriate drug use and responsible self-medication, following practical measures should be implemented in daily practice in community pharmacies:

- Paying attention to patients who seek OTC treatment and do not ask for advice regarding it;
- Starting communication to discover the extent of medication and/or self-medication;
- Offering unsolicited advice as a part of pharmaceutical care;
- If substantial or unnecessary self-medication is discovered, directing patients towards a physician check-up to consider possible deprescription of certain medicines;
- Directing patients who use many concomitant treatments to medication management or pharmacotherapy advisory centers (if available);
- Encouraging patients to perform check-ups of their home pharmacies/medical cabinets and to discard drugs past expiry dates as well as those with unknown purpose at least twice a year;
- Offering patients who might struggle with recognizing potentially toxic drugs to bring their medicines to the pharmacy and help them to separate needed drugs from ones that should be discarded.

If consistently applied in community pharmacies, these measures could result in enhanced health literacy in managing treatments and discarding old drugs and contribute to public health of communities overall.

## 5 Conclusion

The results of this study show that the level of self-medication in the surveyed population is rather high. There is a need to deploy practical interventions to enhance awareness of possible risks and dangers of inappropriate self-medication as well as strengthening education on how to make it responsible. The role of HCPs who are the primary contact for patients should be reinforced especially in communication with the ones who show high inclination towards self-medication. This mostly concerns family physicians and community pharmacists and shows the importance of their mutual collaboration in recognizing the patients’ needs and in guiding patients towards the safe use of drugs. Due to their availability even in underserved neighborhoods, community pharmacists are assumed to be the most available HCPs to guide and advise lay people in matters of proper medication management. The intertwined roles of primary physicians and pharmacists in recognizing irresponsible self-medication practices are crucial for providing adequate and more secure behaviors and patients’ health outcomes. To fully understand the extent of self-medication in various settings as well as diverse population subgroups, it should be further researched.

## Data Availability

The raw data supporting the conclusions of this article will be made available by the authors, without undue reservation.
